# Camel milk consumption patterns and perceptions in the UAE: a cross-sectional study

**DOI:** 10.1017/jns.2022.55

**Published:** 2022-07-21

**Authors:** Leila Cheikh Ismail, Tareq M. Osaili, Maysm N. Mohamad, Hala Zakaria, Aaesha Ali, Asma Tarek, Alizeh Ashfaq, Mohamed A. Al Abdouli, Sheima T. Saleh, Rameez Al Daour, Radhiya AlRajaby, Lily Stojanovska, Ayesha S. Al Dhaheri

**Affiliations:** 1 Department of Clinical Nutrition and Dietetics, College of Health Sciences, University of Sharjah, Sharjah 27272, UAE; 2 Nuffield Department of Women's & Reproductive Health, University of Oxford, Oxford OX1 2JD, UK; 3 Department of Nutrition and Food Technology, Faculty of Agriculture, Jordan University of Science and Technology, Irbid 22110, Jordan; 4 Department of Nutrition and Health, College of Medicine and Health Sciences, United Arab Emirates University, Al Ain 15551, UAE; 5 Technical Services Division, Abu Dhabi Agriculture and Food Safety Authority, Mohamed Bin Zayed City, Abu Dhabi 52105, UAE; 6 Institute for Health and Sport, Victoria University, Melbourne, VIC 14428, Australia

**Keywords:** Food safety, Health benefits, Knowledge, Nutritional value, United Arab Emirates, Unpasteurised milk

## Abstract

Camel milk has been consumed for centuries due to its medicinal and healing properties. The present study aims to investigate the consumption patterns of camel milk and perceived benefits and risks among adults in the United Arab Emirates. A self-administered online questionnaire was developed in English and Arabic languages and was completed online by 852 adults. Socio-demographic characteristics, camel milk consumption patterns and perceived knowledge of the benefits and risks of camel milk were investigated. About 60 % of the participants have tried drinking camel milk, but only a quarter (25⋅1 %) were regular consumers. The most consumed camel milk products after fresh milk were yoghurt and flavoured milk. The most popular additions to camel milk were honey, turmeric and sugar. Most consumers had less than one cup of camel milk per day (57⋅0 %). Camel milk consumers preferred it over other types of milk due to its nutritional value (66⋅4 %) and medicinal properties (39⋅3 %). Among consumers, 58⋅4 % reported consuming unpasteurised camel milk. Reasons included the belief that it is fresher (87⋅2 %), better for the immune system (41⋅6 %), and higher in nutrients (39⋅2 %). Overall, participants had inadequate knowledge about the health benefits of camel milk (7⋅11 ± 5⋅3 out of 25). Males and camel milk consumers had a significantly higher knowledge about the health benefits of camel milk compared to females and non-consumers (*P* < 0⋅05). Although positive perceptions were common, misperceptions appear to be prevalent among non-consumers. Consuming unpasteurised camel milk is a major public health concern, thus national regulations are essential.

## Introduction

Camels play an essential role in the lives of many communities as they were and still are important livelihood assets, especially in arid zones due to their significant adaptations to harsh climates and the provision of milk, meat and means of transportation. In 2020, the Gulf Corporate Council (GCC) camel dairy market reached a value of 502⋅3 million US dollars and it is expected to portray moderate growth of 7⋅1 % by the year 2026^(1)^.

In the past few decades, camel milk has gained widespread market exposure and commercialisation due to its advertised health benefits parallel to the increasing prevalence of non-communicable diseases (NCDs) and the continuously increasing health consciousness of consumers^(1)^. Moreover, the COVID-19 pandemic has proliferated the increasing recognition of camel milk value amid the ongoing pursuit of immune-boosting foods by consumers during the pandemic^(2)^. In the United Arab Emirates (UAE) pasteurised camel milk is regularly sold across the country in many forms (fresh milk, flavoured milk, milk powder, ghee, drinking yoghurt, etc.) and it is exported worldwide. However, camel milk farming is still a common practice in the UAE, thus consumption of unpasteurised camel milk from family-owned farms might be of concern.

Although camel milk contains numerous nutrients that provide health benefits, it may also pose a threat to health in case of contamination. Foodborne illnesses might occur as a result of consuming unpasteurised milk and the most susceptible individuals are infants, children, pregnant women, the elderly and immunocompromised persons^(3)^. In Saudi Arabia and Morocco, analyses of raw camel milk samples indicated contamination due to poor handling practices and hygiene conditions^(4,5)^. Therefore, possible health risks may be associated with the consumption of raw camel milk due to the potential presence of pathogens and their serious adverse health impacts^(6)^. For instance, a brucellosis outbreak was recorded among fourteen individuals in Qatar and was traced back to raw camel milk consumption^(7)^. Nonetheless, the demand for raw milk has increased noticeably in recent years as a growing number of consumers believe that milk pasteurisation strips some of the milk's nutritional value and health benefits^(8)^. Moreover, despite being acknowledgeable of the health hazards of consuming unpasteurised raw milk, people continue its consumption with the belief that the benefits outweigh the risks^(9)^.

Camel milk is historically well-known for its medicinal and healing properties. These properties have been published in limited systematic reviews and were mainly determined in *in vitro* studies^(10)^. The most common benefits of consuming camel milk are those related to diabetes and autism spectrum disorders (ASDs), which have been well-documented in several randomised controlled trials (RCTs)^(11–16)^. Other promising therapeutic properties have been recently published including those related to its antibacterial and antiviral properties^(17,18)^, antioxidant and anticancer effect^(19)^, and hypoallergenic effects^(20)^. Moreover, camel milk has gained popularity as an alternative dairy product for lactose intolerant people^(21,22)^. These benefits are related to a great extent to camel milk's unique composition. A recent systematic review indicated that camel milk is considered superior to cow's milk as it is lower in lactose, saturated fats and cholesterol, higher in unsaturated fatty acids, has ten times more iron, and three times more Vitamin C, and is easier to digest^(23)^. Camel milk is also high in antioxidants and vitamin A, D, E and B vitamins^(23)^. The myriad health benefits of camel milk come with a high price tag and a slightly salty taste compared to cow's milk which might be off-putting for many.

Consumer awareness and knowledge are key in determining the demand for any product. Moreover, attitudes and perceptions towards inherent attributes of products can greatly impact the consumers’ willingness to use or purchase products^(24)^. Literature unravels knowledge gaps about dairy products which have an impact on consumer behaviour in terms of the quantity and quality of milk and dairy consumed^(25)^. In a study investigating consumer consumption and attitude towards cow's milk, an immensely low consumption rate was reported^(26)^. Moreover, multiple barriers to consuming milk were identified among adults including mistaken perceptions about the health benefits and dietary value of milk^(26)^. In addition, a recent study investigated consumers’ acceptance of camel milk before and after knowing its benefits and concluded that awareness of camel milk's health benefits and being prone to NCDs significantly increase willingness to consume camel milk^(24)^.

The literature provides a decent body of evidence on camel milk's nutrition and health benefits; however, limited research is available on the consumption patterns of camel milk. Therefore, the present study aims to examine consumption and consumers’ knowledge and attitudes towards camel milk in the UAE. We hypothesised that consumption of unpasteurised camel milk is common in the UAE and that camel milk consumers are aware of its health benefits.

## Methods

### Study design and participants

This was a population-based cross-sectional study conducted between March and May 2021 among adults in the UAE. The target population included adults who were 18 years and older and living in the UAE. A total of 852 participants were recruited from all seven emirates in the UAE. Participants were recruited by disseminating a web link connecting to the online survey. The link was distributed using e-mail invitations and social media platforms, e.g., LinkedIn™, Facebook™ and WhatsApp™. To minimise selection bias that may arise with snowball sampling (including interrelated-similar individuals), participants were advised to refer to a maximum of three non-family member individuals. Participation was completely voluntary, with no monetary or non-monetary incentives given, and the subjects were free to withdraw at any time. No personal identification data was collected. An information sheet explaining the objective of the study, and the protocol was provided on the first page of the online survey. Consenting subjects were directed to the next part of the study questions.

### Sample size calculation

The sample size was calculated based on the following formula^(27)^:
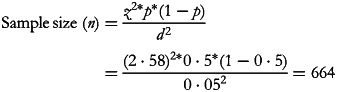
where *Z* = 2⋅58 for a confidence level (*α*) of 99 %, population proportion (*p*) = 50 %, and a margin of error (*d*) = 5 %. Considering a non-respondent rate of 10 %, a minimum of 730 subjects will be required. However, the present study included 852 participants who completed the online survey.

### Ethical statement

This study was conducted according to the guidelines laid down in the Declaration of Helsinki and all procedures involving human subjects were approved by the University of Sharjah Research Ethics Committee (REC-21-03-03-06-S). An electronic informed consent was obtained from all participants.

### Survey questionnaire

A self-administered, multicomponent questionnaire was developed online using Google Forms and was available in English and Arabic languages. The survey draft was developed by the research team to investigate consumption patterns and perceptions regarding camel milk based on the literature^(24,28)^. It was then revised and validated by a panel of experts in the fields of nutrition and food science. Afterwards, the questionnaire was pilot tested with thirty-four people in the UAE, and necessary adjustments in language were made. Data from the pilot testing were not included in the results of this research. The online survey consisted of three main sections and required 15–20 min to complete; (1) socio-demographic data: sex, age, education level, marital status, employment status and having health or nutrition-related qualifications; (2) self-reported camel milk consumption and (3) knowledge and attitude towards benefits and risks of camel milk.

### Camel milk consumption

**Fig. 1.**
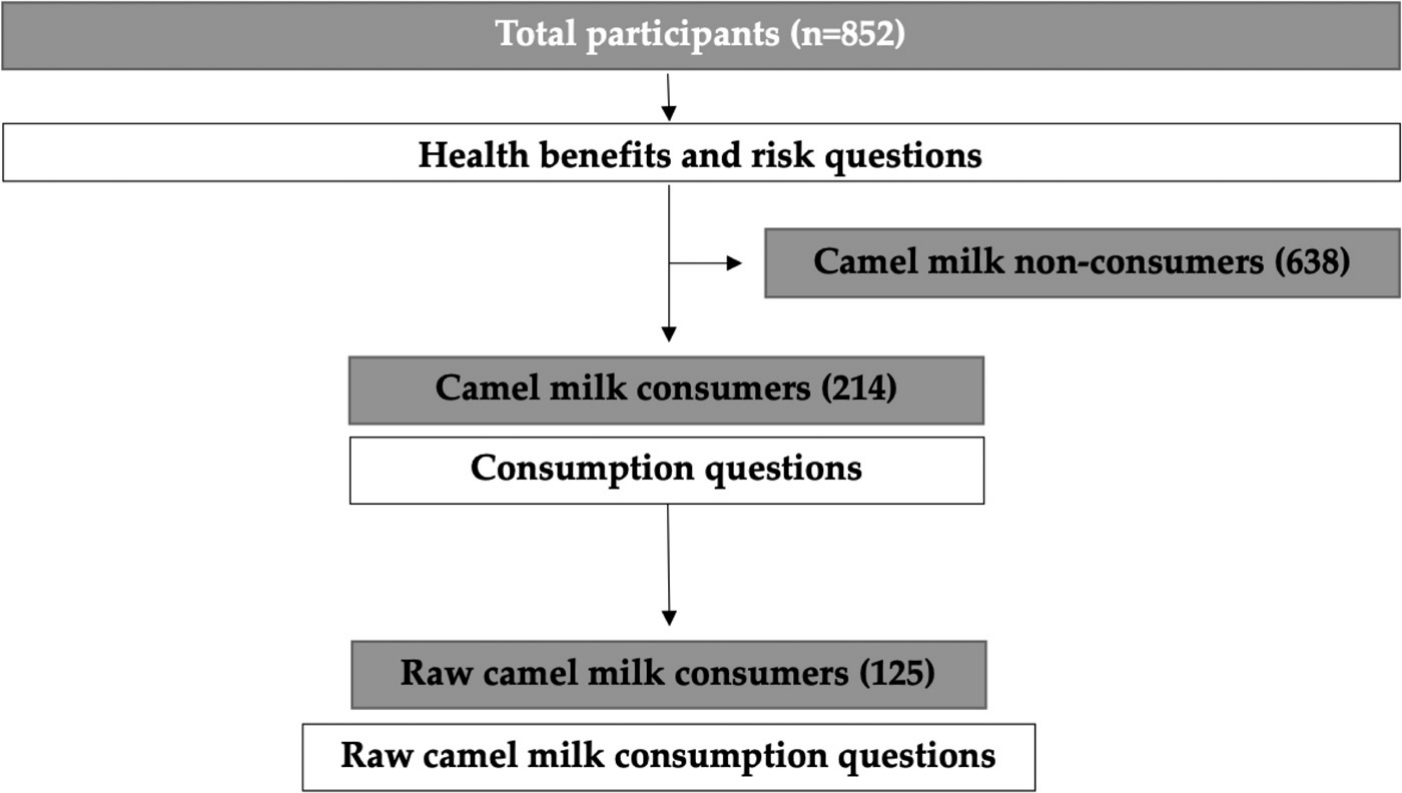
Study population flowchart diagram.

Participants were asked whether they have ever tried consuming camel milk and if they were regular consumers. Only camel milk consumers were able to complete this section, whereas non-consumers were directed to the health benefits and risks section of the survey (Fig. 1).

This section included questions about preferred types of camel milk products, duration of consumption, frequency of consumption and if they add anything to camel milk. Consumers were also asked to identify who mostly consumes camel milk in their household, if they consume it as treatment or prevention of a certain disease, the reason for preferring camel milk over other types of milk, and where they obtain camel milk from. Unpasteurised camel milk consumption was also investigated. Consumers were asked if they consume raw camel milk, perceived advantages and disadvantages of raw milk, and if they have ever experienced any food poisoning symptoms upon consumption.

### Health benefits and risk

This section measured knowledge and attitudes towards camel milk benefits and risks among all study participants. The knowledge part included twenty-five questions and the response options were ‘Yes’, ‘No’ and ‘Not sure’. Each question had one correct answer and the knowledge score was derived out of twenty-five for each participant based on the total of correct answers.

The attitudes section comprised eleven items. Participants were asked if they were concerned about the fat, cholesterol, sugar, sodium and pathogens content of camel milk. They were also asked if they were concerned about the taste, shelf-life, price, safety and availability of camel milk. Response options included ‘Agree’, ‘Neutral’ and ‘Disagree’.

### Statistical analysis

Statistical Package for the Social Sciences (SPSS) version 26.0 (IBM, Chicago, IL, USA) was used to analyse the collected data. Continuous data were expressed as mean ± standard deviation (sd), and categorical data were expressed as counts and percentages. A knowledge score ranging from 0 to 25 was derived for each participant based on the number of correct answers to health knowledge questions. An independent *t*-test and one-way ANOVA test were used to determine the association between demographic variables and knowledge score. The *χ*^2^ test was used to compare the association of agreement with attitudes statements between camel milk consumers and non-consumers. The *P*-values at <0⋅05 were considered statistically significant.

## Results

### Socio-demographic information

A total of 852 participants completed the survey. The socio-demographic characteristics of the study population are presented in Table 1. The mean age of the surveyed participants was 33⋅9 ± 12⋅5 years. More females than males completed the online survey (24⋅5 % males). Almost three out of four participants had a bachelor's degree or higher. Most participants were married (54⋅8 %), worked full-time (42⋅5 %) and did not have health-related qualifications (88 %).

**Table 1.** Socio-demographic characteristics of participants (*n* 852)

Variables	*n*	%
Gender		
Females	643	75⋅5
Males	209	24⋅5
Age (years)		
18–25	311	36⋅5
26–35	189	22⋅2
36–45	175	20⋅5
>46	177	20⋅8
Education level		
Less than a bachelor's degree	201	23⋅6
Bachelor's degree or above	651	76⋅4
Marital status		
Single	385	45⋅2
Married	467	54⋅8
Employment status		
Full-time	362	42⋅5
Part-time	30	3⋅5
Student	246	28⋅9
Unemployed	144	16⋅9
Retired	70	8⋅2
Have health-related qualifications		
Yes	102	12
No	750	88

### Camel milk consumption

Almost two-thirds (59⋅5 %) of the participants reported drinking camel milk at least once and a quarter of them (25⋅1 %) were regular consumers. Participants who reported consuming camel milk were asked about their consumption habits (Table 2). Camel milk consumers reported consuming mostly fresh milk followed by yoghurt and flavoured milk (88⋅8, 17⋅3, and 10⋅3 %, respectively). Over one-third of participants reported consuming camel milk for more than 20 years (31⋅8 %) and half of them reported not adding anything to their milk (50⋅0 %). However, honey, turmeric and sugar were the most popular add-ons to camel milk, respectively. Camel milk was consumed within the household mostly by adults (53⋅7 %). Almost half of camel milk consumers purchase their milk from family farms and do not consume it for medical reasons.

**Table 2.** Camel milk consumption habits among participants who reported drinking camel milk (*n* 214)

Variables	*N*	%
What camel milk products do you consume?^a^		
Fresh milk	190	88⋅8
Dried milk	6	2⋅8
Flavoured milk	22	10⋅3
Yoghurt	37	17⋅3
How many years have you been consuming camel milk?		
Less than 1 year	23	10⋅7
1–5 years	50	23⋅4
5–10 years	38	17⋅8
10–20 years	35	16⋅4
More than 20 years	68	31⋅8
Do you add anything when drinking camel milk?^a^		
No	107	50⋅0
Honey	68	31⋅8
Water	6	2⋅8
Turmeric	29	13⋅6
Sugar	25	11⋅7
Camel urine	1	0⋅5
Ginger	8	3⋅7
Fenugreek	5	2⋅3
Saffron	2	0⋅9
Coffee	4	1⋅9
Who mainly consumes camel milk in your household?^a^		
Infants	16	7⋅5
Children	32	15⋅0
Young adults	63	29⋅4
Adults	115	53⋅7
Elderly	81	37⋅9
All members	61	28⋅5
Where do you usually purchase camel milk from?		
Supermarket	78	36⋅4
Family farm	110	51⋅4
Farmer's market	26	12⋅1
Do you consume camel milk as a treatment/prevention of certain conditions?		
Yes	62	29⋅0
No	152	71⋅0

a Multiple responses were allowed.

**Fig. 2.**
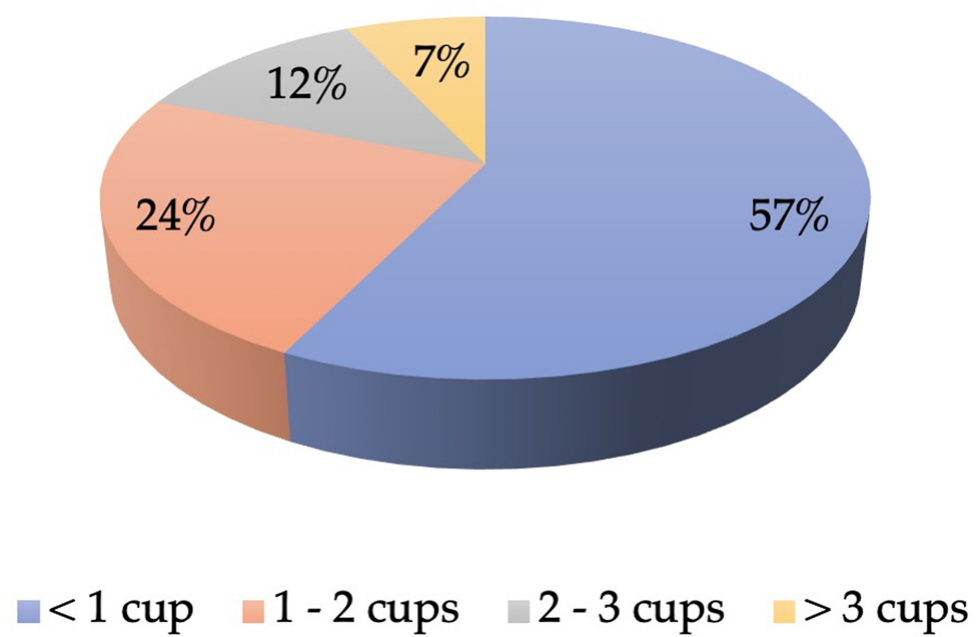
Percentage of daily consumption of camel milk among camel milk consumers (*n* 214).

The daily consumption of camel milk was calculated based on reported frequency and consumption by consumers as presented in Fig. 2. Most consumers had less than one cup (<250 ml) of camel milk per day (57⋅0 %).

**Fig. 3.**
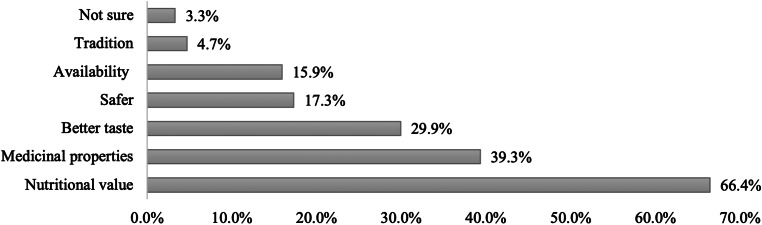
Consumers’ reasons for preferring camel milk over other types of milk (*n* 214). Multiple responses were allowed.

Most consumers preferred camel milk compared to other types of milk due to its nutritional value (66⋅4 %), followed by its medicinal properties (39⋅3 %) and taste (29⋅9 %) (Fig. 3).

**Fig. 4.**
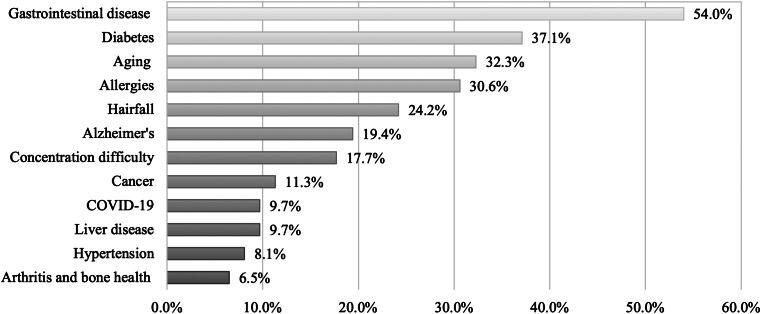
Use of camel milk for medical reasons or as a treatment for certain diseases (*n* 62). Multiple responses were allowed.

Almost one-third of camel milk consumers reported using camel milk for medical reasons or as a treatment for certain diseases (Fig. 4). Most of them used camel milk as a treatment for gastrointestinal diseases (54 %), followed by diabetes (37⋅1 %), aging (32⋅3 %) and allergies (30⋅6 %). Interestingly, around 10 % reported using camel milk for cancer, COVID-19, liver disease and hypertension.

### Consumption of unpasteurised camel milk

A total of 125 (58⋅4 %) camel milk consumers reported consuming unpasteurised camel milk (Table 3). Most of the participants believed that unpasteurised milk is fresher (87⋅2 %), better for the immune system (41⋅6 %) and higher in nutrients (39⋅2 %). Most raw camel milk consumers knew that consuming unpasteurised milk increases the risk of foodborne disease (44⋅8 %) but over a third of them believed that there are no drawbacks to drinking unpasteurised camel milk (36⋅0 %). Moreover, only 10⋅4 % of them reported experiencing food poisoning symptoms after consuming unpasteurised camel milk.

**Table 3.** Unpasteurised camel milk consumption habits among participants who reported drinking raw camel milk (*n* 125)

Variables	*n*	%
Do you drink raw camel milk?		
Yes	125	58⋅4
No	89	41⋅6
Why do you consume unpasteurised camel milk instead of pasteurised?^a^		
Fresher	109	87⋅2
Better support for the immune system	52	41⋅6
Higher in nutrient	49	39⋅2
It tastes better than pasteurised milk	45	36⋅0
Better protection against diseases	36	28⋅8
It can be easily digested	32	25⋅6
Less chemicals	23	18⋅4
It may help protect against asthma and allergies	22	17⋅6
Support local farmers	20	16⋅0
Less of an environmental impact	16	12⋅8
What do you think are the main disadvantages of unpasteurised camel milk?^a^		
Risk of foodborne disease	56	44⋅8
Poor shelf-life	34	27⋅2
No drawbacks	45	36⋅0
Not sure	32	25⋅6
Did you ever experience any food poisoning symptoms (nausea, vomiting, diarrhoea, abdominal pain, etc.) after drinking unpasteurised camel milk?		
Yes	13	10⋅4
No	108	86⋅4
Not sure	4	3⋅2

a Multiple responses were allowed.

### Health and nutritional benefits

The mean knowledge score of the participants was 7⋅1 ± 5⋅3 (28⋅4 %). Table 4 shows the association between the mean knowledge scores and different socio-demographic variables. Findings indicate a significant difference between female and male knowledge, where males scored higher compared to females (*P* < 0⋅001). Moreover, camel milk consumers had significantly higher scores compared to non-consumers (*P* < 0⋅001).

**Table 4.** Knowledge score of health and nutritional benefits of camel milk among participants (*n* 852)

Variables	Mean (sd) Score^a^	*P*-value*
Gender		
Females	6⋅7 (5⋅3)	<0⋅001
Males	8⋅3 (5⋅2)	
Age (years)		
18–25	7⋅1 (5⋅5)	0⋅793
26–35	7⋅3 (5⋅5)	
36–45	6⋅8 (4⋅7)	
>46	7⋅3 (5⋅3)	
Education level		
Less than Bachelor's	7⋅2 (4⋅6)	0⋅790
Bachelor's or above	7⋅1 (5⋅5)	
Marital status		
Single	7⋅3 (5⋅5)	0⋅385
Married	7⋅0 (5⋅1)	
Employment status		
Full-time	7⋅2 (5⋅3)	0⋅374
Part-time	7⋅8 (5⋅1)	
Student	7⋅5 (5⋅4)	
Unemployed	6⋅6 (5⋅4)	
Retired	6⋅4 (4⋅5)	
Have health-related qualifications		
Yes	7⋅1 (5⋅5)	0⋅954
No	7⋅1 (5⋅3)	
Camel milk consumers		
Yes	8⋅9 (4⋅6)	<0⋅001
No	6⋅5 (5⋅4)	

a Range: 0–25.

* The *P*-values indicate the statistical significance of the independent *t*-test and one-way ANOVA test.

Table 5 shows the proportion of participants who agreed with attitude statements, as well as a comparison between camel milk consumers and non-consumers. Among camel milk consumers, almost two-thirds of the participants believed that camel milk contains beneficial active components, and one-third of them believed that camel milk is not readily available and expensive. By contrast, almost half of non-consumers were concerned about the taste of camel milk, and over a third of them were concerned with the pathogens content of unpasteurised camel milk, its price, availability and thought that camel milk contains active components that reduce the risk of diseases.

**Table 5.** Percentage agreement with attitudes statements, comparison between camel milk consumers (*n* 214) and non-consumers (*n* 638)

	*n* (%)		
Attitude/belief	Consumers (*n* 214)	Non-consumers (*n* 638)	*P*-value* (two-sided)
I am concerned with the fat content of camel milk	18 (8⋅4)	131 (20⋅5)	<0⋅001
I am concerned with the cholesterol content of camel milk	18 (8⋅4)	138 (21⋅6)	<0⋅001
I am concerned with sugar content of camel milk	21 (9⋅8)	90 (14⋅1)	<0⋅001
I am concerned with sodium content of camel milk	19 (8⋅9)	92 (14⋅4)	<0⋅001
I am concerned with pathogens content of unpasteurised camel milk	31 (14⋅5)	238 (37⋅3)	<0⋅001
I am concerned with taste of camel milk	23 (10⋅7)	315 (49⋅4)	<0⋅001
Camel milk spoils easily	33 (15⋅4)	108 (16⋅9)	0⋅010
Camel milk is expensive	68 (31⋅8)	227 (35⋅6)	<0⋅001
Camel milk is not readily available	75 (35⋅0)	277 (43⋅4)	<0⋅001
Unpasteurised camel milk is not recommended for pregnant women, children and elderly	54 (25⋅2)	250 (39⋅2)	<0⋅001
Camel milk contains active components that reduce the risk of diseases	128 (59⋅8)	277 (43⋅4)	<0⋅001

* The *P*-values indicate the statistical significance of the *χ*^2^ test.

## Discussion

The present study is the first to assess consumption patterns of camel milk and perceived benefits and risks among the adult population of the UAE. One-quarter of the participants were regular camel milk consumers. Overall, the findings of the present study revealed that camel milk consumption is relatively low in the UAE despite its high availability in the market. This could be related to the high price of camel milk compared to cow's milk and concerns about its taste. As a litre of camel milk costs on average in the UAE around 6 $ US, while a litre of cow's milk is about 1⋅5 $ US. Moreover, results showed that most of the participants have tried camel milk at least once. This can be due to the increasing interest in the therapeutic and nutritional value of camel milk^(29)^ as well as growing consumer enthusiasm towards camel milk during the COVID-19 pandemic^(2)^.

In the present study, camel milk consumers reported mostly drinking fresh milk compared to other products such as fermented, flavoured or dried. This is expected as research shows that most camel milk-producing countries consume it in the liquid form^(29)^. Moreover, the daily consumption rate was assessed, and most consumers reported having less than one cup of camel milk per day followed by consuming one to two cups daily. These findings are in line with previous findings from the NHANES 2007–2010 data in the USA^(30)^ that showed the daily consumption of milk in adults was below the recommendations of two to three cups per day^(31)^. The present study also showed that camel milk was a popular choice for adults and was rarely given to infants and children. This could be related to the belief of camel milk consumers that camel milk is not safe for children. However, a systematic review of the nutritional and therapeutic virtues of camel milk concluded that camel milk is safe for children and could be used as an effective treatment for autistic children^(32)^. Furthermore, most consumers purchase camel milk from a family farm as it could be more affordable and convenient; however, regulations on the processing and handling of farm camel milk are lacking.

The present study also investigated consumers’ motives for preferring camel milk. Most consumers drink camel milk for its nutritional value, followed by its medicinal properties. Of those using camel milk for disease prevention and treatment, most consumers reported using it as a treatment for gastrointestinal diseases. Limited robust data is available on such relation; however, consistent with our finding a systematic review revealed that due to the high anti-inflammatory protein content in camel milk, it could have a positive impact on the stomach and other gastrointestinal disorders^(33)^. Moreover, as camel milk contains less lactose than cow's milk, it can be better tolerated by people suffering from lactose intolerance^(22)^. Almost a third of consumers reported using camel milk for diabetes, allergies and aging. The effect of camel milk in improving glycemic control in diabetic patients and reducing the incidence of developing diabetes is well documented by RCTs^(11–14,34,35)^. Other data have shown that camel milk can benefit those with cow's milk allergies since it lacks β-lactoglobulin and a particular β-casein, which are the two components in cow's milk that cause allergies^(23)^. However, no sound data is available on its use as an anti-aging component.

Drinking raw or unpasteurised camel milk has been an integrated traditional belief for the people of the UAE. However, a large body of strong evidence confirms that raw milk is not recommended for consumption due to increased risks of foodborne diseases^(36,37)^. In the present study, an alarming proportion of consumers reported drinking raw camel milk without heat treatment. This could be attributed to the fact that most of them conveniently obtain the milk from their family/friend's farm, making it less likely for them to do any treatment for the milk. Raw camel milk advocates in this study believed that unpasteurised milk is fresher, higher in nutrients and better for the immune system compared to pasteurised milk. A systematic review and meta-analysis of forty studies indicated that pasteurisation causes only minor insignificant losses of water-soluble vitamins which are already low in the milk and can be easily obtained from other dietary sources^(38)^. Another study supported this finding as it indicated that there is no nutritional advantage of raw milk over pasteurised milk^(39)^. Moreover, the heating of raw milk does not change the nutritional value or the perceived benefits of consuming raw milk^(40)^.

Most raw milk consumers in this study reported disadvantages to raw milk such as the increased risk of foodborne illness and poor shelf-life, while around a third of them believed that there are no disadvantages to consuming raw milk. These results are comparable to community research in England about raw cow milk consumption as respondents reported drawbacks such as the risk of disease or infection, poor shelf-life and a comparatively lesser proportion believed that there are no drawbacks^(41)^. Another surprising finding was that most of the participants did not report experiencing any food poising symptoms after drinking unpasteurised camel milk. An explanation for this might be due to confusion about the meaning of the term pasteurised as conveyed in the England raw milk drinking reports^(41)^.

The present study measured the objective knowledge of the health and nutrition aspects of camel milk. Overall, participants exhibited a low level of knowledge about camel milk although most of them consume camel milk for its health and nutrition values. This could imply the need for further investigation on sources of health-related information as unreliable sources may lead to various misconceptions and improper practices. Furthermore, associations between the knowledge scores and socio-demographic characteristics were tested, wherein results showed that regular consumption was the strongest predictor of a higher level of knowledge. This is supported by findings from a study about liquid milk where consumers who exhibit higher knowledge tend to buy and consume more functional foods^(42)^. In the present study, non-consumers had more misconceptions and concerns about camel milk compared to consumers. Several studies indicated that consumers’ beliefs and attitude predict their purchase intent and consumption^(26,42)^. This agrees with the results from our study as camel milk consumers had a significantly more positive attitude towards camel milk compared to non-consumers. Consumers were far less concerned with the nutritive composition of camel milk and held better beliefs regarding camel milk's beneficial active compounds than non-consumers, emphasising their higher level of knowledge about camel milk. On the other hand, non-consumers were more concerned about the taste of camel milk and the pathogen content of raw camel milk. This could be a reasonable concern as camel milk possesses a sharp, tangy, and salty taste compared to cow's milk^(17)^. Furthermore, the cost of camel milk was of concern to both groups as well. A recent systematic review on consumers’ willingness to pay for organic food concluded that consumers are mostly driven by factors such as environmental concern, health awareness and locality^(43)^. This could apply to our sample as consumers persist in camel milk consumption even though it costs three times more than cow's milk, due to their perceptions and knowledge about camel milk and support for local farms and local products.

Limitations of the present study include the use of a web-based, self-reported questionnaire which could lead to sampling bias and response bias because only those who have access to the internet and have social media accounts were able to participate. Furthermore, the cross-sectional design and convenience sampling may result in a less representative sample of the population. Despite these limitations, the present study has several strengths. This is the first study that assesses the knowledge, attitude and consumption patterns of camel milk in the UAE. Moreover, the methodological design facilitated a wide range of data collection in the country. In addition, the present study identified knowledge gaps about camel milk and provided insights into current consumption patterns and attitudes towards camel milk in the UAE.

## Conclusion

Although positive perceptions about the health benefits of camel milk were common among participants that consume it, misperceptions appear to be prevalent among non-consumers. Moreover, consumption of unpasteurised camel milk was prevalent, which is a major public health concern requiring national regulations to ensure the safety of consumers.
